# Association between urinary biomarkers of total sugars intake and measures of obesity in a cross-sectional study

**DOI:** 10.1371/journal.pone.0179508

**Published:** 2017-07-19

**Authors:** Rachel Campbell, Natasha Tasevska, Kim G. Jackson, Virag Sagi-Kiss, Nick di Paolo, Jennifer S. Mindell, Susan J. Lister, Kay-Tee Khaw, Gunter G. C. Kuhnle

**Affiliations:** 1 Department of Food & Nutritional Sciences, University of Reading, Reading, United Kingdom; 2 School of Nutrition and Health Promotion, Arizona State University, Phoenix, Arizona, United States of America; 3 NatCen Social Research, London, United Kingdom; 4 Research Department of Epidemiology & Public Health, UCL, London, United Kingdom; 5 Institute of Biological, Environmental and Rural Sciences, University of Aberystwyth, Aberystwyth, United Kingdom; 6 Department of Public Health and Primary Care, University of Cambridge, Cambridge, United Kingdom; McMaster University, CANADA

## Abstract

Obesity is an important modifiable risk factor for chronic diseases. While there is increasing focus on the role of dietary sugars, there remains a paucity of data establishing the association between sugar intake and obesity in the general public. The objective of this study was to investigate associations of estimated sugar intake with odds for obesity in a representative sample of English adults. We used data from 434 participants of the 2005 Health Survey of England. Biomarkers for total sugar intake were measured in 24 h urine samples and used to estimate intake. Linear and logistic regression analyses were used to investigate associations between biomarker-based estimated intake and measures of obesity (body mass intake (BMI), waist circumference and waist-to-hip ratio) and obesity risk, respectively. Estimated sugar intake was significantly associated with BMI, waist circumference and waist-to-hip ratio; these associations remained significant after adjustment for estimated protein intake as a marker of non-sugar energy intake. Estimated sugar intake was also associated with increased odds for obesity based on BMI (OR 1.02; 95%CI 1.00–1.04 per 10g), waist-circumference (1.03; 1.01–1.05) and waist-to-hip ratio (1.04; 1.02–1.06); all OR estimates remained significant after adjusting for estimated protein intake. Our results strongly support positive associations between total sugar intake, measures of obesity and likelihood of being obese. It is the first time that such an association has been shown in a nationally-representative sample of the general population using a validated biomarker. This biomarker could be used to monitor the efficacy of public health interventions to reduce sugar intake.

## Introduction

Dietary sugars, in particular free sugars (according to the WHO definition monosaccharides and disaccharides added to foods and beverages by the manufacturer, cook or consumer, and sugars naturally present in honey, syrups, fruit juices and fruit juice concentrates”[1]) have received increasing attention from the WHO [1] as well as the UK government [2] and the UK’s Scientific Advisory Committee on Nutrition (SACN) [3]. While sugar intake is often associated with an increased risk of obesity [4], the evidence available from observational studies is more ambiguous and shows significant associations for sugar-sweetened beverages (SSB) [5,6] only, but fails to show consistent associations for intake of sugars as nutrients [6–9]. However, in most observational studies, sugar intake was assessed using self-reported data. It is likely that this has introduced bias, especially as underreporting of diet has been found to be more prevalent among obese people [10–12] and it is sugar-rich foods that are most commonly underreported [13]. It is possible that reporting bias contributes to the observed inverse associations between sugar intake and BMI [4]. Moreover, the random error associated with self-reported dietary data is likely to attenuate observed associations.

Urinary sugars have been investigated [14,15] and validated [16,17] as dietary biomarkers of total sugars (i.e., the sum of intrinsic, milk and free sugars) and sucrose [18] in both normal weight and obese individuals [19], and can help to resolve the discrepancy between self-reported and actual intake. This biomarker relies on the total excretion of sucrose and fructose within 24h and therefore requires complete 24h urine samples. While we have been able to show a positive association between the biomarker measured in spot urines and BMI, and risk of overweight and obesity [4,20], the lack of validation data on the performance of sucrose and fructose as dietary biomarkers from spot urines makes these results more difficult to interpret and weakens the utility of this biomarker.

In this study, we have used exclusively biomarker data to estimate sugar intake as self-reported dietary data were not available. This allowed us not only to investigate associations between sugar intake and obesity risk, but also to test the feasibility of applying this biomarker to an existing cohort as an instrument to help monitor consumption and to investigate associations between sugar intake and obesity.

## Method

### Study population

The Health Survey for England is a health examination survey of nationally-representative samples of the general population. A new, random, household-based sample has been selected annually since 1991. Individuals living at the selected private addresses are recruited to the study, answer a questionnaire through face-to-face interview, and have trained interviewers measure height and weight. Nurses take other physical measurements and collect biological samples [21]. The measurement of height, weight (interviewer), and waist and hip circumference (nurse) followed the protocols of the 2003 Health Survey for England [22]. No data on diet, except for fruit and vegetable intake, were collected by interview. Ethical approval was obtained from the London Multi-centre Research Ethics Committee (MREC).

We used data of participants from the 2005 Health Survey for England (HSE 2005) with the aim of obtaining a nationally-representative sample of the general population aged 19 to 64 years living in England [22]. As a supplement to the main HSE 2005, the *English FSA Dietary Sodium Study (EFSAUS)*, a sub-sample of adult participants were asked to provide a 24-hour urine sample to measure urinary sodium. Overall, 498 survey participants (200 men, 298 women, Table 1), aged 19 and over, who provided a 24-hour urine sample were identified and included in the study. Data collection took place between October 2005 and July 2006, with the majority of fieldwork being completed by March 2006.

**Table 1:** Study population characteristics and description of analytical sample. Median and inter-quartile range or absolute number and proportion. See S1 Table for more details.

	Women		Men	
n	261	247^†^	173	165^†^
Age [years]	45 (36–55)	44 (36–55)	48 (37–56)	48 (36–55)
Waist circumference [cm]	85.6 (78.5–94. 6)	85.6 (78.8–94.5)	97.9 (90.4–107)	97.7 (90.4–107)
Waist-to-hip ratio	0.81 (0.77–0.86)	0.81 (0.77–0.86)	0.93 (0.89–0.97)	0.93 (0.89–0.98)
BMI [kg/m^2^]	26.0 (23.5–29.8)	26.0 (23.5–29.7)	27.5 (25.3–30.4)	27.3 (25.2–30.1)
Normal weight	108 (41%)	101 (41%)	33 (22%)	38 (23%)
Overweight	93 (36%)	91 (37%)	86 (50%)	84 (51%)
Obese	60 (23%)	55 (22%)	49 (28%)	43 (26%)
Urinary excretion				
Sucrose [mg/d]	26.4 (11.6–50.6)	25.1 (10.7–46.1)	38.6 (23.9–62.6)	37.2 (23.0–59.7)
Fructose [mg/d]	18.1 (9.4–33.3)	17.5 (9.2–29.8)	18.4 (11.7–27.1)	18.1 (11.1–26.3)
Nitrogen [g/d]	10.3 (8.0–12.3)	10.4 (8.0–12.3)	13.3 (10.4–16.4)	13.3 (10.5–16.4)
Estimated intake				
Total Sugars [g/d]	127 (66.1–219)	117 (62.0–201)	167 (93.4–247)	162 (91–227)
Protein [g/d]	79.4 (61.8–94.8)	80.0 (62.0–94.7)	102 (80.4–127)	102 (80.6–127)

^†^excluding the top 5% of estimated total sugar intake

### 24-hour urine collection

Participants were asked to collect all urine they passed during a 24-hour period starting from the second morning urine void of the 24-hour collection day, and ending with the first urine void the following morning. P-amino-benzoic acid (PABA) was used to test for completeness of 24h urine collection and only complete samples (with >85% PABA recovery in urine) were used for this analysis [23]. All samples were stored at -20°C until analysis (2006 to 2013).

### Analysis of urinary sucrose and fructose

Urine samples were thawed at room temperature, centrifuged to remove protein aggregates and analysed using an ILAB600 clinical chemistry analyser (Werfen (UK) Limited, Warrington) with a sucrose, fructose and glucose enzyme kit (Sucrose/D-Glucose/D-Fructose; Boehringer Mannheim, R-Biopharm, Enzymatic BioAnalysis/Food Analysis, Darmstadt, Germany). This method determines D-glucose by measuring NADPH + H^+^ formation following phosphorylation of D-glucose by hexokinase and subsequent oxidation by NADPH^+^-dependent glucose-6-phosphate dehydrogenase. NADPH + H^+^ is determined by changes in absorption at 340 nm. Sucrose and D-fructose are determined indirectly following the conversion of D-fructose into D-glucose by phosphoglucose-isomerase or β-fructosidase and calculating the difference in D-glucose concentration before and after conversion. The concentration range for sucrose and D-fructose was 2.5 to 200 mg/L, for D-glucose it was 2.5 to 150 mg/L; samples exceeding these concentrations were diluted 1 in 10 with purified water and reanalysed. The intra-assay CV for a 25 mg/L glucose quality control (QC) sample was less than 2% and the inter-assay CV was 3.6%. The inter-assay CV was also determined for fructose and sucrose and found to be less than 7%. All concentrations measured were above the lower-limit of quantification. 24-h urinary sucrose and fructose were calculated based on urinary fructose and sucrose concentration (mg/L) and 24-h urine volume.

### Analysis of urinary nitrogen

We measured 24-h urinary nitrogen, a recovery biomarker for protein intake, to partialy control for non-sugars energy intake. Urine samples were thawed at room temperature prior to analysis. Approximately 1 ml of samples was weighed into a tin foil capsule. For Total Nitrogen (N %) determination, the sample was combusted in oxygen and the nitrogen released measured with a thermal conductivity cell using a LECO FP-428 Analyser (LECO Corp., St. Joseph, MI). The coefficients of variation for within-run and within-laboratory precision were 1.8 and 3.8%, respectively for an internal quality control sample containing 1% N. The limit of quantification for the test was 0.018% N.

### Biomarker-based estimates of total sugars and protein intake

Estimated total sugars intake was calculated based on a calibration equation for the sugars biomarker developed from a feeding study with participants consuming their usual diet under highly controlled conditions conducted in the UK [16], which describes the association between the biomarker and true intake [17]

$$CM_{i,j}=M_{i,j}-1.67-0.02\times S_{i}+0.71\times A_{i}$$

where CM is log transformed calibrated biomarker of person i at time point j, i.e. predicted total sugars intake, M is log transformed sum of 24-hour urine fructose and sucrose, S is sex (male: S = 0, female: S = 1) and A is log transformed age. Given that the feeding study was based on participants’ usual diet, the calibration equation developed in the study could be applied to free-living UK individuals, however, with the assumption that the parameters of the equation remain stable within a population. Estimated protein intake was calculated based on the assumptions that 81% of dietary nitrogen is recovered from urine [24] and an average nitrogen content of proteins is 16% [P: protein intake (g/d), N: total nitrogen excretion (g/d)]:

$$P=\frac{N}{0.81}\times 6.25$$

### Data handling

Calculated fructose and sucrose concentrations of zero were assigned a value of 0.1 to allow for a log_2_-transformation of the data.

### Statistical analyses

All data were processed using R version 3.3.2 [25]. Biomarker estimates of total sugars and protein intakes were normally distributed and used without transformation. We used unadjusted models to investigate associations between estimated total sugars intake and BMI and obesity risk (based on WHO definition [26] either as BMI ≥ 30 kg/m^2^, waist-to-hip ratio > 0.85 for women and > 0.90 for men, or waist circumference of 80 cm for women and 94 cm for men), given the calibration equation for the sugars biomarker which we used to estimate total sugars intake included age and sex.

Urinary sugars (fructose, sucrose and their sum) were skewed to the right and log_2_-transformed, as were the ratios of urinary sugars to urinary nitrogen and estimated sugar intake to estimated protein intake. We used the ratios of estimated total sugars to protein intake or urinary sugars to urinary nitrogen to investigate the effect of sugars while controlling for dietary composition. Models with uncalibrated urinary fructose, uncalibrated urinary sucrose or estimated protein intake and BMI and obesity risk were adjusted for age and sex. Associations with BMI were investigated using linear regression models; OR for obesity (as estimate of risk) was estimated using logistic regression. Urinary nitrogen or estimated protein intake was included in the models to control for protein intake as a contributor to energy intake. P<0.05 was used as threshold for statistical significance.

## Results

### Study population

Study population characteristics and description of the analytical sample are shown in Table 1 and S1 Table. Complete data on age, sex, BMI, waist-to-hip-ratio and 24h urine volume were available for 298 women and 200 men (n = 498). Due to missing samples or insufficient volume, not all samples could be analysed for urinary biomarkers; data on urinary sugars and nitrogen are available for 261 women and 173 men only (n = 434).

The distribution of estimated dietary sugar intake (median 144 g/d, range 0–2777 g/d) was skewed right with some extremely high values. We have therefore truncated the data at the 95^th^ centile of estimated intake (527 g/d). The remaining sample included 247 women and 165 men (n = 411). Participants in the top 5^th^ centile (14 women, 8 men) were older (mean age 50.6 years *vs* 44.8 years, *t*-test: p = 0.024) and had a higher excretion of sucrose (247 mg/d *vs* 36.4 mg/d, *t*-test: p<0.001) and fructose (84.9 mg/d *vs* 22.3 mg.d, *t*-test: p<0.001) than those in the remaining sample. There were however no statistically significant differences in BMI, waist circumference, waist-to-hip ratio or protein intake.

### Associations between estimated intake, measures of obesity and odds for obesity

**Fig 1:**
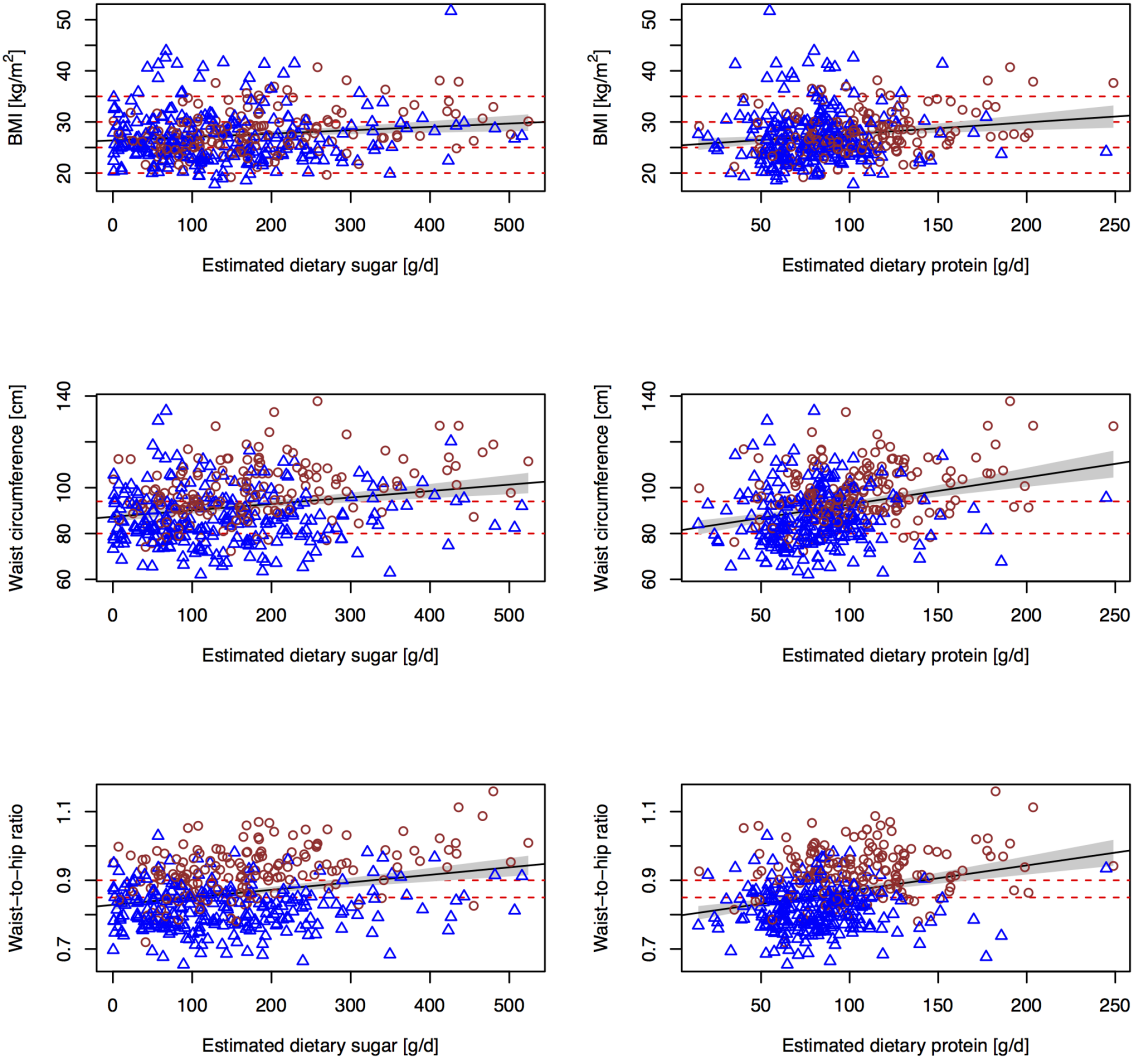
Associations between estimated sugars and protein intake and obesity markers. Associations between estimated sugars and protein intake and BMI, waist circumference and waist-to-hip ratio in men (brown circles) and women (blue triangles).

Estimated total sugars and protein intake were positively associated with BMI, waist circumference and waist-to-hip ratio, both independently (Table 2) and when combined in the same model (Table 3 and Fig 1). They were also positively associated with odds for obesity when using waist-to-hip-ratio as the obesity marker. However, associations were weaker for BMI and waist circumference. Significant associations were observed only for estimated protein intake (both independently and in the combined model, using estimated sugar and protein intake) and estimated sugar intake when using BMI as the obesity marker, and only for estimated sugar intake in the combined model when using waist circumference. In Supplemental material, we report findings on uncalibrated 24-h urinary sucrose and fructose, and 24-h urinary protein, rather than biomarker-based estimates of sugars and protein intake, respectively, in relation to BMI, waist circumference, waist-to-hip ratio (S2 Table) and odds for obesity (S3 Table).

**Table 2:** Associations between biomarker estimated total sugars and protein intake and BMI (β and 95% CI per 10 g) and odds for obesity risk (OR and 95% CI per 10 g) for each compound intake independently (univariate models).

	Regression coefficient (β and 95% CI per 10 g/d increase)			OR for obesity^‡^ (95% CI) per 10 g/d increase		
	BMI	Waist circumference	Waist-to-hip ratio [× 100]	BMI	Waist circumference	Waist-to-hip ratio [× 100]
Total estimated sugars intake	0.066 (0.024; 0.108)^††^	0.281 (0.165; 0.396)^†††^	0.219 (0.145; 0.292)^†††^	1.02 (1.00; 1.04)^†^	1.03 (1.01; 1.05) ^††^	1.04 (1.02; 1.06)^†††^
Estimated protein intake	0.229 (0.095; 0.365)^†††^	1.180 (0.818; 1.543)^†††^	0.745 (0.511; 0.979)^†††^	1.08 (1.02; 1.15)^†^	1.05 (0.99; 1.12)	1.12 (1.05; 1.19) ^†††^

^†^p<0.05;

^††^p<0.01’

^†††^p<0.001;

^‡^ BMI ≥ 30 kg/m^2^; waist circumference > 85 cm (women) or 94 cm (men); waist-to-hip ratio > 0.85 (women) or 0.90 (men)

**Table 3:** Associations between biomarker estimated total sugars and protein intake and BMI (β and 95% CI per 10 g) and odds for obesity risk (OR and 95% CI per 10 g) in a multivariate model, including estimated sugars and protein intake.

	Regression coefficient (β and 95% CI per 10 g/d increase)			OR for obesity^‡^ (95% CI) per 10 g/d increase		
	BMI	Waist circumference	Waist-to-hip ratio [× 100]	BMI	Waist circumference	Waist-to-hip ratio [× 100]
Total estimated sugars intake	0.055 (0.012; 0.097)^†^	0.220 (0.106; 0.333) ^†††^	0.182 (0.109; 0.254)^†††^	1.02 (1.00; 1.04)	1.03 (1.01; 1.05)^††^	1.03 (1.01; 1.05)^†††^
Estimated protein intake	0.197 (0.061; 0.333)^††^	1.049 (0.686; 1.412)^†††^	0.636 (0.404; 0.868)^†††^	1.07 (1.01; 1.14)^†^	1.03 (0.97; 1.10)	1.10 (1.03; 1.17)^††^

^†^p<0.05;

^††^p<0.01’

^†††^p<0.001;

^‡^ BMI ≥ 30 kg/m^2^; waist circumference > 85 cm (women) or 94 cm (men); waist-to-hip ratio > 0.85 (women) or 0.90 (men)

### Associations between ratio of sugar-to-protein intake, BMI and odds for obesity

We have used the ratio of estimated total sugar and protein intake as a surrogate marker of dietary composition, in particular the contribution of sugars to total energy intake (Table 4). Except for a positive association with waist-to-hip ratio, non were statistically significant. However, we found a positive association for 24-h urinary sucrose to nitrogen ratio in relation to BMI, waist circumference, waist-to-hip ratio and odds for obesity measured by waist circumference and waist-to-hip ratio (S4 Table).

**Table 4:** Associations between the ratio of estimated sugars and protein intake, and BMI (β and 95% CI) and odds for obesity (OR and 95% CI). Estimates in each column represent a separate model. Data for urinary sugars and nitrogen are shown in S4 Table.

	Linear regession Regression coefficient(β and 95% CI) ^‡^		
	BMI [kg/m^2^]	Waist circumference [cm]	Waist-to-hip ratio [× 100]
Ratio estimated sugars and protein intake	0.108 (-0.187; 0.403)	0.628 (-0.191; 1.448)	0.596^†^ (0.070; 1.122)
	Logistic regression OR for Obesity^❡^ (95% CI)		
	BMI ≥ 30 kg/m^2^	Waist circumference > 85 cm (women) or 94 cm (men)	Waist-to-hip ratio > 0.85 (women) or 0.90 (men)
Ratio estimated sugars and protein intake	1.01 (0.88; 1.17)	1.08 (0.95; 1.22)	1.08 (0.96; 1.23)

^†^ p<0.05;

^‡^log_2_ transformed and adjusted for age and sex;

^❡^BMI ≥ 30 kg/m2; waist circumference > 85 cm (women) or 94 cm (men); waist-to-hip ratio > 0.85 (women) or 0.90 (men)

## Discussion

In this study, we have used exclusively biomarker and biomarker-based estimates of intake and not self-reported data to investigate associations between sugar intake and odds for obesity. In our study population, using biomarker-based intake estimates, sugars were significantly associated with BMI, waist circumference and waist-to-hip ratio, and these associations remained significant after adjustment for biomarker-based protein intake. Estimated sugars intake was also associated with increased odds for obesity as measured by BMI, waist-circumference and waist-to-hip ratio. The association between sugar intake and obesity risk in the general public is difficult to investigate because of the known limitation of self-reported dietary assessment, in particular the tendency to underreport the intake of perceived unhealthy foods and foods with high sugar content, especially among overweight individuals [11]. Indeed, observational studies relying on self-reported intake have long produced inconsistent results and generated controversy and consistent data are available only for an association between obesity and sugar-sweetened beverages [5,6] but not total sugar intake [9]. It is important to investigate this further to establish the role of sugar, but also of other nutrients.

Biomarker estimated protein intake was similar, although slightly higher, than in the 2008/9 UK National Diet and Nutrition Survey (NDNS), a representative national dietary survey using self-reported (food diaries) dietary data (80 g/d *vs* 66 g/d for women, 102 g/d *vs* 89 g/d for men) [27]. In contrast, biomarker estimated sugar intake was considerably higher than intakes reported in the NDNS (117 g/d *vs* 78 g/d for women, 162 g/d *vs* 107 g/d for men). One explanation for these findings is that sugar intake, but not protein intake, is often underreported [13].

Our data showed a significant association between biomarker-estimated total sugar intake and both measures of obesity and likelihood of being obesity, confirming positive associations between total sugar intake, measures of obesity and odds of obesity. The main strengths of this study are that the samples are from a study designed to be representative of the English population and that 24h urine samples were available for more than 85% of the study population (S1 Table for details). Moreover, the calibration equation that was used to calibrate the biomarker and generate estimate of sugars intake was developed in a UK feeding study under a UK diet. Limitations of the study include the small sample size, the cross-sectional study design and the use of multiple comparisons without adjustment; many associations were of borderline statistical significance and a larger study would allow further exploration. A further limitation is the absence of objectively measured energy intake, for example using double-labelled water [28].

There was no information about stomach ulcers—which increase gastrointestinal permeability for (unhydrolized) sucrose—or impaired kidney function, which could affect urinary fructose and sucrose excretion. Previous research has shown that neither obesity nor stomach ulcers have a significant impact on the biomarker used [19,20], but there is a paucity of data investigating the effect of impaired renal function. As sucrose is excreted rapidly and almost completely in urine [29], it is unlikely that diabetic kidney disease affects urinary sucrose concentrations. The physiological processes are more complex for fructose as it involves active reabsorption in the kidney [30] and higher urinary fructose concentrations have been observed in patients with diabetes [31], although it is not clear whether this is due to impaired kidney function. Increased excretion would result in an overestimation of sugar intake in participants with impaired kidney function, but there is currently no information to what extend fructose excretion is affected by kidney impairment. Estimates for the prevalence of moderate to severe chronic kidney disease (CKD) from the US suggest that it is approximately 5% [32].

While BMI is commonly used to diagnose obesity, there are some limitations due to its inability to discriminate between fat and lean mass [33]. We have therefore also included waist circumference and waist-to-hip ratio in our analyses and the results are comparable. Indeed, associations between estimated sugar intake and odds for obesity are stronger when using waist-circumference and waist-to-hip ratio as measures of obesity.

**Fig 2:**
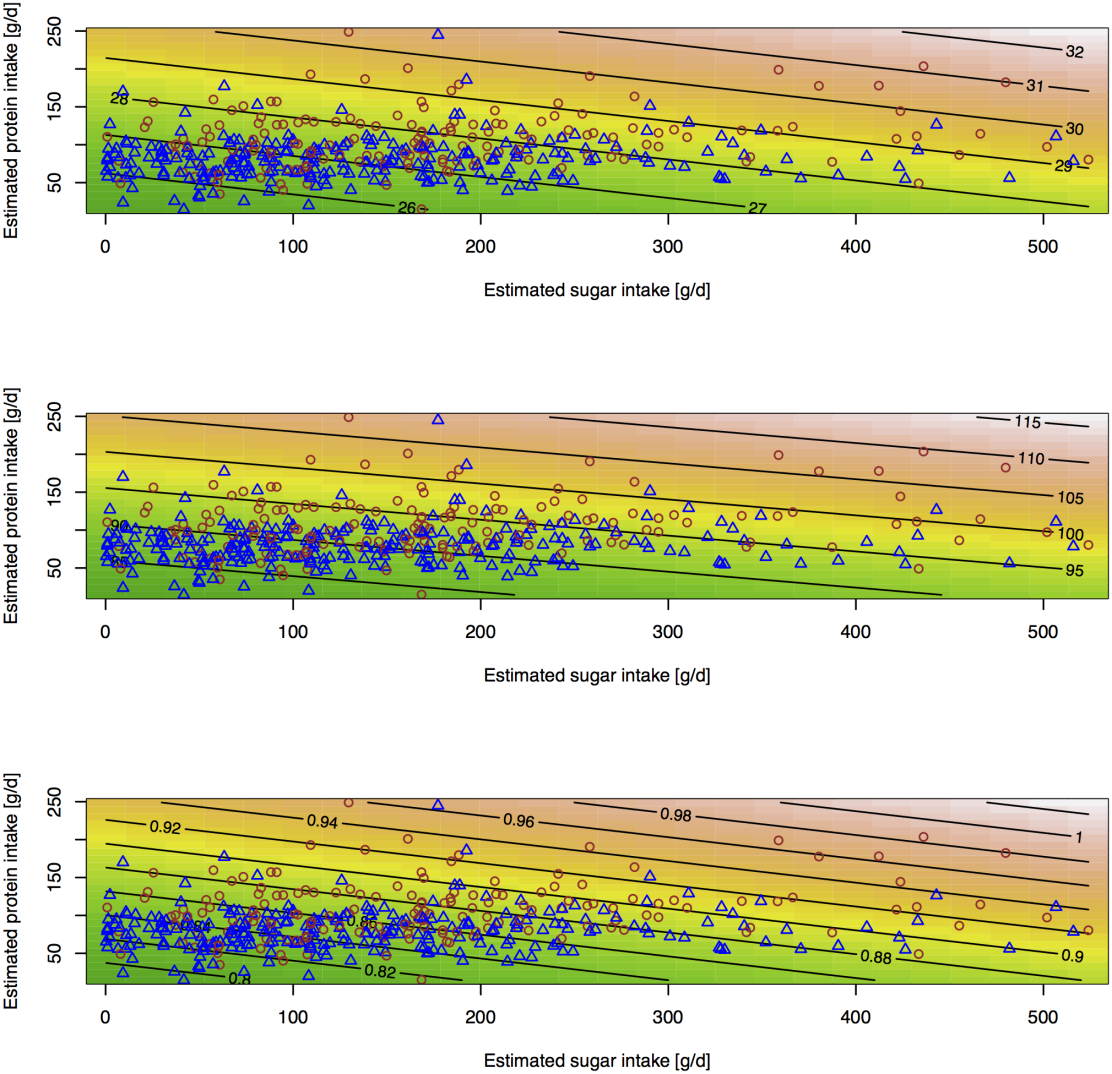
Association between estimated sugar, protein intake and obesity risk markers using a response surface model. Association between estimated total sugars and protein intake and (a) BMI [kg/m^2^], (b) waist circumference [cm] and (c) waist-to-hip ratio in women (blue triangles) and men (brown circles) using a response surface model. Points show data for individual participants, contour lines and colours estimated BMI, waist circumference and waist-to-hip ratio of linear regression mode respectively.

A possible explanation for the association between estimated sugar intake and measures of obesity could be that sugar intake is simply acting as a marker of total energy intake. Protein is currently the only macronutrient for which there is a reliable recovery biomarker [12,24], and total energy intake can only be estimated using double-labelled water [28,34]. In the UK, protein intake contributed approximately 15% to 20% of total daily energy intake [27], and we have therefore used biomarker-estimated protein intake as a surrogate marker of total energy intake. Independently, estimated protein intake was also associated with BMI, waist-circumference and waist-to-hip ratio, and odds for obesity based on BMI and waist-to-hip ratio. These associations remained significant when combining sugar and protein in the same model, although both became slightly attenuated (Fig 2).

If estimated sugar intake was only a marker of total energy intake, a considerably stronger attenuation of the observed associations would have been expected. There are however several limitations to this approach that need to be taken into consideration when interpreting the results. Dietary data is likely more variable and a wider range of protein intake can be expected. A larger random variability would only attenuate observed associations, but a systematic variability can introduce bias and data available do not allow us to predict whether obese participants are more likely to increase [13] or decrease [35] relative protein intake. Another limitation are the physiological processes underlying urinary nitrogen as biomarker of protein intake. In participants gaining weight, this marker underestimates total protein intake and therefore attenuates the observed observation. For participants actively gaining weight, the role of sugar intake is therefore likely overestimated, and due to the cross-sectional design of the study it is not possible to identify these participants.

We have explored these relationships further by using uncalibrated biomarker data (i.e., 24-h urinary sucrose and fructose and 24-h urinary nitrogen) (S2–S4 Tables). Our data show a strong association between urinary sucrose and measures of obesity, as well as the odds of oobesity based on waist circumference and waist-to-hip ratio. These associations were generally strengthened when including sucrose and fructose in the same model. Conversely, there were no significant associations for urinary fructose and only few associations were significant for total urinary sugars.

These results suggest that the associations between sugar intake and measures of obesity are mainly driven by sucrose. In contrast to fructose, which is derived from dietary fructose and hydrolysed sucrose and extensively metabolised, the only source of urinary sucrose is dietary sucrose [14–16,36], making it more sensitive to changes in sucrose intake, the main contributor to intake of free sugars in the UK. Furthermore, high-fructose corn syrup (HFCS) or isoglucose was not commonly used in England at the time of the study as import and production was tighly controlled as part of the European Union sugar regime (Commission Regulation (EC) No 314/2002). Therefore the main source of dietary fructose were fruit and fruit products, such that fructose was most likely a surrogate marker of their intake.

Our results show that urinary sugars can be used to estimate sugar intake in the general population when 24h urine samples are available. In the context of current discussions regarding sugar intake and the recently updated WHO recommendations on sugars intake [1], the biomarker could be used to monitor the efficacy of public health interventions, although it would be important to take other sources of sugars, such as fruits, into consideration. Furthermore, we showed significant associations between sugar intake and BMI, confirming results of previous observations in EPIC Norfolk [4,20]. It is the first time that such an association has been shown in a nationally-representative sample of the general population using a validated biomarker. Our data also show significant associations between protein intake and measures of obesity and odds for being obesie, however, in contrast to protein, sucrose is not an essential part of the human diet and intake can be reduced without adverse effects.

## Supporting information

S1 Table Study population characteristics and description of analytical sample. Median and inter-quartile range or absolute number and proportion.
(DOC)

S2 Table Associations between 24h excretion of sucrose, fructose and nitrogen and BMI, waist-circumference and waist-to-hip-ratio (β and 95% CI). Data were log_2_-transformed and models are adjusted for age and sex. Estimates in each column represent a separate model.
(DOC)

S3 Table Associations between 24h uriny excretion of sucrose, fructose and nitrogen and and odds for obesity (OR and 95% CI). Data were log_2_-transformed and models are adjusted for age and sex. Estimates in each column represent a separate model.
(DOC)

S4 Table Associations between ratio of sugars and protein intake, and ratio of urinary sugars and nitrogen and BMI (β and 95% CI) and obesity risk (OR and 95% CI). Estimates in each column represent a separate model.
(DOC)
