# Supplementary material for: Association between urinary biomarkers of total sugars intake and measures of obesity in a cross-sectional study
Source: PLoS One. 2017 Jul 19;12(7):e0179508. doi: 10.1371/journal.pone.0179508 (PMC5517003; doi:10.1371/journal.pone.0179508)
Supplement: S1 Table — Median and inter-quartile range or absolute number and proportion. (DOC) [file pone.0179508.s001.doc]

S1Table: Study population characteristics and description of analytical sample. Median and inter-quartile range or absolute number and proportion.

|  | Women | | | | Men | | | |
| --- | --- | --- | --- | --- | --- | --- | --- | --- |
| n | 298 | 285 | 261 | 247 | 200 | 189 | 173 | 165 |
| Available data |  |  |  |  |  |  |  |  |
| Age, Sex, BMI, hip-to-waist ratio and urine volume | X | X | X | X | X | X | X | X |
| Urinary sugars |  | X | X | X |  | X | X | X |
| Urinary nitrogen |  |  | X | X |  |  | X | X |
| Excluding extremes^†^ |  |  |  | X |  |  |  | X |
| Age [years] | 45 (34 – 56) | 45 (36 – 54) | 45 (36 – 55) | 44 (36 – 55) | 48 (37 – 56) | 48 (37 – 56) | 48 (37 – 56) | 48 (36 – 55) |
| Waist circumference [cm] | 85.6 (78.7 – 94.5) | 85.6 (78.9 – 94.5) | 85.6 (78.5 – 94. 6) | 85.6 (78.8 – 94.5) | 98.4 (90.9 – 108) | 98.4 (91.4 – 108) | 97.9 (90.4 – 107) | 97.7 (90.4 – 107) |
| Waist-to-hip ratio | 0.82 (0.77 – 0.86) | 0.82 (0.77 – 0.86) | 0.81 (0.77 – 0.86) | 0.81 (0.77 – 0.86) | 0.93 (0.89 – 0.98) | 0.93 (0.89 – 0.98) | 0.93 (0.89 – 0.97) | 0.93 (0.89 – 0.98) |
| BMI [kg/m^2^] | 25.6 (23.1 – 29.4) | 25.5 (23.0 – 29.5) | 26.0 (23.5 – 29.8) | 26.0 (23.5 – 29.7) | 27.2 (24.5 – 30.2) | 27.2 (24.5 – 30.1) | 27.5 (25.3 – 30.4) | 27.3 (25.2 – 30.1) |
| Normal weight | 131 (44%) | 125 (44%) | 108 (41%) | 101 (41%) | 56 (28%) | 52 (28%) | 33 (22%) | 38 (23%) |
| Overweight | 103 (35%) | 99 (35%) | 93 (36%) | 91 (37%) | 90 (45%) | 87 (46%) | 86 (50%) | 84 (51%) |
| Obese | 64 (22%) | 61 (21%) | 60 (23%) | 55 (22%) | 54 (27%) | 50 (27%) | 49 (28%) | 43 (26%) |
| Urinary excretion |  |  |  |  |  |  |  |  |
| Sucrose [mg/d] | — | 26.0 (12.0 – 50.3) | 26.4 (11.6 – 50.6) | 25.1 (10.7 – 46.1) | — | 38.6 (23.8 – 62.7) | 38.6 (23.9 – 62.6) | 37.2 (23.0 – 59.7) |
| Fructose [mg/d] | — | 17.7 (9.3 – 32.5) | 18.1 (9.4 -33.3) | 17.5 (9.2 – 29.8) | — | 17.7 (9.3 – 32.5) | 18.4 (11.7 – 27.1) | 18.1 (11.1 – 26.3) |
| Nitrogen [g/d] | — | — | 10.3 (8.0 – 12.3) | 10.4 (8.0 – 12.3) | — | — | 13.3 (10.4 – 16.4) | 13.3 (10.5 – 16.4) |
| Estimated intake |  |  |  |  |  |  |  |  |
| Total Sugars [g/d] | — | 122 (66.1 – 216) | 127 (66.1 – 219) | 117 (62.0 – 201) | — | 168 (91.3 – 247) | 167 (93.4 – 247) | 162 (91 – 227) |
| Protein [g/d] | — | — | 79.4 (61.8 – 94.8) | 80.0 (62.0 – 94.7) | — | — | 102 (80.4 – 127) | 102 (80.6 – 127) |

^†^excluding the top 5% of estimated total sugar intake
