# Supplementary material for: Association between urinary biomarkers of total sugars intake and measures of obesity in a cross-sectional study
Source: PLoS One. 2017 Jul 19;12(7):e0179508. doi: 10.1371/journal.pone.0179508 (PMC5517003; doi:10.1371/journal.pone.0179508)
Supplement: S2 Table — Data were log2-transformed and models are adjusted for age and sex. Estimates in each column represent a separate model. (DOC) [file pone.0179508.s002.doc]

S2 Table: Associations between 24h excretion of sucrose, fructose and nitrogen and BMI, waist-circumference and waist-to-hip-ratio (β and 95% CI). Data were log2-transformed and models are adjusted for age and sex. Estimates in each column represent a separate model.

| Regression coefficient (β and 95% CI per doubling of excretion) | | | | | | | | | | | | | | | | |  |
| --- | --- | --- | --- | --- | --- | --- | --- | --- | --- | --- | --- | --- | --- | --- | --- | --- | --- |
|  | | | BMI [kg/m^2^] | | | | | | | | | | | | | |  |
| Sum of 24-h urinary sucrose and fructose [mg/d] | | | 0.201  (-0.099; 0.501) | | — | — | — | — | 0.171  (-0.129; 0.471) | | — | | — | | — | |  |
| 24-h urinary sucrose [mg/d] | | | — | | 0.243  (0.029; 0.458)^†^ | — | 0.309  (0.073; 0.545)^†^ | — | — | | 0.236  (0.023; 0.450)^†^ | | — | | 0.318  (0.083; 0.553)^††^ | |  |
| 24-h urinary fructose [mg/d] | | | — | | — | -0.030  (-0.258; 0.199) | -0.166  (-0.416; 0.083) | — | — | | — | | -0.067  (-0.296; 0.163) | | -0.209  (-0.460; 0.042) | |  |
| 24-h urinary Nitrogen [g/d] | | | — | | — | — | — | 0.960  (0.077; 1.842)^†^ | 0.910  (0.024; 1.797)^†^ | | 0.931  (0.052; 1.810)^†^ | | 0.997  (0.105; 1.890)^†^ | | 1.038  (0.151; 1.925)^†^ | |  |
|  | | | Waist circumference [cm] | | | | | | | | | | | | | |  |
| Sum of 24-h urinary sucrose and fructose [mg/d] | | 0.754  (0.022; 1.486)^†^ | | — | | — | — | — | | 0.666  (-0.066; 1.397) | | — | | — | | — |  |
| 24-h urinary sucrose [mg/d] | | — | | 0.782  (0.259; 1.305)^† †^ | | — | 0.946  (0.370; 1.521)^††^ | — | | — | | 0.762  (0.242; 1.282)^††^ | | — | | 0.973  (0.402; 1.544)^†††^ |  |
| 24-h urinary fructose [mg/d] | | — | | — | | 0.004  (-0.556; 0.563) | -0.415  (-1.024; 0.194) | — | | — | | — | | -0.105  (-0.666; 0.456) | | -0.540  (-1.150; 0.070) |  |
| 24-h urinary Nitrogen [g/d] | | — | | — | | — | — | 2.857  (0.702; 5.012)^† †^ | | 2.665  (0.506; 4.825)† | | 2.764  (0.626; 4.901)^††^ | | 2.916  (0.736; 5.096)^††^ | | 3.041  (0.887; 5.196)^††^ |  |
|  | | | Waist-to-hip ratio [× 100] | | | | | | | | | | | | | |  |
| Sum of 24-h urinary sucrose and fructose [mg/d] | 0.474  (0.081; 0.867)^†^ | | | — | | — | — | — | | 0.458  (0.063; 0.853)^††^ | — | | | — | | — | |
| 24-h urinary sucrose [mg/d] | — | | | 0.415  (0.133; 0.696)^††^ | | — | 0.496  (0.186; 0.805)^††^ | — | | — | 0.411  (0.129; 0.692)^††^ | | | — | | 0.502  (0.192; 0.812)^††^ | |
| 24-h urinary fructose [mg/d] | — | | | — | | 0.015  (-0.286; 0.316) | -0.204  (-0.532; 0.123) | — | | — | — | | | -0.008  (-0.312; 0.296) | | -0.233  (-0.564; 0.098) | |
| 24-h urinary Nitrogen [g/d] | — | | | — | | — | — | 0.627  (-0.540; 1.794) | | 0.495  (-0.672; 1.662) | 0.577  (-0.581; 1.734) | | | 0.632  (-0.549; 1.813) | | 0.697  (-0.472; 1.865) | |

^†^p<0.05; ^††^p<0.01’ ^††^p<0.001
