# Supplementary material for: Association between urinary biomarkers of total sugars intake and measures of obesity in a cross-sectional study
Source: PLoS One. 2017 Jul 19;12(7):e0179508. doi: 10.1371/journal.pone.0179508 (PMC5517003; doi:10.1371/journal.pone.0179508)
Supplement: S3 Table — Data were log2-transformed and models are adjusted for age and sex. Estimates in each column represent a separate model. (DOC) [file pone.0179508.s003.doc]

S3 Table: Associations between 24h uriny excretion of sucrose, fructose and nitrogen and and odds for obesity (OR and 95% CI). Data were log_2_-transformed and models are adjusted for age and sex. Estimates in each column represent a separate model.

|  | Obesity risk (OR and 95% CI – per doubling of excretion) | | | | | | | | |
| --- | --- | --- | --- | --- | --- | --- | --- | --- | --- |
|  | BMI ≥ 30 kg/m^2^ | | | | | | | | |
| Sum of 24-h urinary sucrose and fructose [mg/d] | 1.04  (0.90; 1.22) | — | — | — | — | 1.03  (0.89; 1.21) | — | — | — |
| 24-h urinary sucrose [mg/d] | — | 1.08  (0.97; 1.22) | — | 1.12  (0.98; 1.27) | — | — | 1.08  (0.97; 1.22) | — | 1.12  (0.99; 1.28) |
| 24-h urinary fructose [mg/d] | — | — | 0.99  (0.89; 1.10) | 0.94  (0.84; 1.07) | — | — | — | 0.97  (0.87; 1.09) | 0.93  (0.82; 1.05) |
| 24-h urinary Nitrogen [g/d] | — | — | — | — | 1.53  (0.97; 2.48) | 1.52  (0.96; 2.46) | 1.53  (0.97; 2.48) | 1.56  (0.99; 2.53) | 1.60  (1.00; 2.61) |
|  | Waist circumference > 85 cm (women) or 94 cm (men) | | | | | | | | |
| Sum of 24-h urinary sucrose and fructose [mg/d] | 1.04 (0.90; 1.22) | — | — | — | — | 1.13  (0.99; 1.30) | — | — | — |
| 24-h urinary sucrose [mg/d] | — | 1.12  (1.02; 1.24) ^†^ |  | 1.16  (1.04; 1.29) ^†^ | — | — | 1.12  (1.02; 1.24) ^†^ | — | 1.16  (1.04; 1.30) ^†^ |
| 24-h urinary fructose [mg/d] | — | — | 0.99  (0.89; 1.10) | 0.93  (0.82; 1.04) | — | — | — | 0.97  (0.87; 1.09) | 0.91  (0.80; 1.03) |
| 24-h urinary Nitrogen [g/d] | — | — | — | — | 1.43  (0.97; 2.15) | 1.38  (0.93; 2.07) | 1.41  (0.95; 2.12) | 1.46  (0.98; 2.19) | 1.49  (0.99; 2.25) |
|  | Waist-to-hip ratio > 0.85 (women) or 0.90 (men) | | | | | | | | |
| Sum of 24-h urinary sucrose and fructose [mg/d] | 1.07  (0.93; 1.24) | — | — | — | — | 1.07  (0.93; 1.24) | — | — | — |
| 24-h urinary sucrose [mg/d] | — | 1.11  (1.00; 1.24) | — | 1.15  (1.03; 1.30) ^†^ | — | — | 1.11  (1.00; 1.24) | — | 1.16  (1.03; 1.31) ^†^ |
| 24-h urinary fructose [mg/d] | — | — | 0.97  (0.87; 1.08) | 0.91  (0.80; 1.03) | — | — | — | 0.97  (0.87; 1.08) | 0.90  (0.80; 1.02) |
| 24-h urinary Nitrogen [g/d] | — | — | — | — | 1.09  (0.71; 1.68) | 1.08  (0.70; 1.66) | 1.09  (0.71; 1.67) | 1.11  (0.72; 1.71) | 1.13  (0.74; 1.76) |

^†^p<0.05
