# Supplementary material for: Association between urinary biomarkers of total sugars intake and measures of obesity in a cross-sectional study
Source: PLoS One. 2017 Jul 19;12(7):e0179508. doi: 10.1371/journal.pone.0179508 (PMC5517003; doi:10.1371/journal.pone.0179508)
Supplement: S4 Table — Estimates in each column represent a separate model. (DOC) [file pone.0179508.s004.doc]

S4 Table: Associations between ratio of sugars and protein intake, and ratio of urinary sugars and nitrogen and BMI (β and 95% CI) and obesity risk (OR and 95% CI). Estimates in each column represent a separate model.

|  |  | Regression coefficient(β and 95% CI) ^‡^ | | | | | Obesity risk^❡^ (OR and 95% CI) ^‡^ | | | | |
| --- | --- | --- | --- | --- | --- | --- | --- | --- | --- | --- | --- |
|  |  | BMI [kg/m^2^] | | | | | BMI ≥ 30 kg/m^2^ | | | | |
| Estimated intake | Total Sugars/Protein | 0.108  (-0.187; 0.403) | — | — | — | — | 1.01  (0.88; 1.17) | — | — | — | — |
| 24h excretion in urine | Sum sucrose and fructose/Nitrogen | — | 0.087  (-0.206; 0.380) | — | — | — | — | 1.00  (0.87; 1.15) | — | — | — |
|  | Sucrose/Nitrogen | — | — | 0.178  (-0.032; 0.389) | — | 0.265  (0.032; 0.497)^†^ | — | — | 1.05  (0.95; 1.17) | — | 1.08  (0.96; 1.23) |
|  | Fructose/Nitrogen | — | — | — | -0.094  (-0.323; 0.135) | -0.218  (-0.470; 0.035) | — | — | — | 0.96  (0.87; 1.07) | 0.93  (0.82; 1.05) |
|  |  | Waist circumference | | | | | Waist circumference > 85 cm (women) or 94 cm (men) | | | | |
| Estimated intake | Total Sugars/Protein | 0.628  (-0.191; 1.448) | — | — | — | — | 1.08  (0.95; 1.22) | — | — | — | — |
| 24h excretion in urine | Sum sucrose and fructose/Nitrogen | — | 0.406  (-0.311; 1.124) | — | — | — | — | 1.09  (0.95; 1.25) | — | — | — |
|  | Sucrose/Nitrogen | — | — | 0.586  (-0.072; 1.100) | — | 0.811  (0.243; 1.379)^††^ | — | — | 1.10  (0.99; 1.21) | — | 1.14  (1.02; 1.27) ^†^ |
|  | Fructose/Nitrogen | — | — | — | -0.187  (-0.748; 0.374) | -0.566  (-1.183; 0.050) | — | — | — | 0.96  (0.86; 1.07) | 0.91  (0.80; 1.02) |
|  |  | Waist-to-hip ratio [× 100] | | | | | Waist-to-hip ratio > 0.85 (women) or 0.90 (men) | | | | |
| Estimated intake | Total Sugars/Protein | 0.596  (0.070; 1.122) | — | — | — | — | 1.08  (0.96; 1.23) | — | — | — | — |
| 24h excretion in urine | Sum sucrose and fructose/Nitrogen | — | 0.384  (0.000; 0.769) | — | — | — | — | 1.06  (0.92; 1.22) | — | — | — |
|  | Sucrose/Nitrogen | — | — | 0.362  (0.085; 0.637) ^†^ | — | 0.457  (0.151; 0.762)^††^ | — | — | 1.10  (0.99; 1.22) | — | 1.14  (1.02; 1.29) ^†^ |
|  | Fructose/Nitrogen | — | — | — | -0.027  (-0.329; 0.275) | -0.240  (-0.572; 0.091) | — | — | — | 0.96  (0.86; 1.08) | 0.90  (0.80; 1.02) |

^†^ p<0.05; ^‡^log_2_ transformed and adjusted for age and sex; ^‡^ ^❡^BMI ≥ 30 kg/m2; waist circumference > 85 cm (women) or 94 cm (men); waist-to-hip ratio > 0.85 (women) or 0.90 (men)
